# Supplementary material for: Complexome profiling of the Chlamydomonas psb28 mutant reveals TEF5 as an early PSII assembly factor
Source: Plant Cell. 2025 Mar 17;37(6):koaf055. doi: 10.1093/plcell/koaf055 (PMC12164590; doi:10.1093/plcell/koaf055)
Supplement: koaf055_Supplementary_Data [file koaf055_supplementary_data.zip › psb28_interactive.zip!/plots/intensity/qualityPlots/peptides/NSS3_Cre13.g573900.t1.2.html]

### Cre13.g573900.t1.2

Trivial name: NSS3  
SeqCoverage [median]: 8.70  
Mass: 76.64 kDa  
  
 

| Sequence | MissedCleavage | Mass [Da] | wtIntensity | muIntensity | Score |
| --- | --- | --- | --- | --- | --- |
| GIEAPELPPVPVDAAGHHTIPAGHHAGEQYSAFVPR | 0 | 3723.85 | 0.00 | 2375.45 | 30.24 |
| GYFTMSQVLFDR | 0 | 1462.69 | 1787.11 | 5733.18 | 127.93 |
| TTAAATMFPHLTAR | 0 | 1487.76 | 6002.17 | 16171.15 | 116.19 |
|  |  |  | 7789.3 | 24279.8 |  |
